# Vision Loss and Recovery after Baerveldt Aqueous Tube Shunt Implantation

**DOI:** 10.1155/2017/4140305

**Published:** 2017-01-18

**Authors:** Esther Lee Kim, Jeffrey Tran, Marc Töteberg-Harms, Jasdeep Chahal, Douglas Rhee, Vikas Chopra, Brian Francis

**Affiliations:** ^1^University of Southern California Eye Institute, Department of Ophthalmology, Keck School of Medicine, The University of Southern California, Los Angeles, CA, USA; ^2^Massachusetts Eye and Ear Infirmary, Department of Ophthalmology, Harvard Medical School, Boston, MA, USA; ^3^Doheny Eye Institute, David Geffen School of Medicine, University of California, Los Angeles, Los Angeles, CA, USA; ^4^Department of Ophthalmology, University Hospital Zurich, Zurich, Switzerland; ^5^Department of Ophthalmology and Visual Sciences, Case Western Medical Center, Cleveland, OH, USA

## Abstract

This study aims to determine the course of vision loss after Baerveldt aqueous tube shunt placement and identify risk factors associated with unexplained severe long-term vision loss, or snuff-out. We retrospectively reviewed 247 eyes of 222 patients who underwent Baerveldt implantations at one of two academic institutions. Postoperative vision loss at 6 months following surgery was categorized as mild-to-moderate versus severe and long-term versus transient. Long-term vision loss, defined as 3 or more lines of Snellen visual acuity (VA) loss compared with preoperative VA, occurred in 63 of 247 eyes (25.5%), and 39 had mild-to-moderate and 24 had severe loss. Of these 63 eyes, 18 had no identifiable cause of vision loss. On multivariate analysis, poorer Snellen VA on postoperative day 1 (POD1) was found to be a significant risk factor for long-term vision loss (*p* = 0.005). In addition, the negative change in preoperative versus POD1 Snellen VA (*p* = 0.021) and the presence of split fixation involving the inferonasal quadrant on preoperative Humphrey visual field (*p* = 0.044) were significant risk factors for snuff-out. Transient vision loss occurred in 76 of 242 eyes (30.8%). In conclusion, vision loss is not uncommon after Baerveldt surgery, with snuff-out occurring in 2.4% of cases in this study.

## 1. Introduction

Glaucoma drainage implants have become an increasingly popular surgical option in cases of refractory glaucoma or failed previous trabeculectomies [1]. Their use may be expanding to primary surgical management in patients with complex or congenital glaucoma and even in more traditional cases with a high risk of trabeculectomy failure. Glaucoma drainage devices have been shown to effectively lower intraocular pressure (IOP) to levels similar to that after trabeculectomy and can thus reduce progression of glaucomatous visual field loss. However, drainage devices are not without known complications, including accelerated corneal endothelial damage, hypotony, tube or plate erosion, strabismus, and infection.

Graefe was the first to report that central vision may be compromised soon after surgery in chronically glaucomatous eyes with reduced visual fields [2, 3]. We explored this phenomenon of “snuff-out,” or long-term severe unexplained vision loss, after trabeculectomy and found a 2% prevalence in the study population [4]. Furthermore, snuff-out was significantly associated with preoperative split fixation of visual fields, preoperative number of quadrants with split fixation, and the occurrence of postoperative choroidal effusions, even after resolution.

However, no prior studies have explored the phenomenon of snuff-out after aqueous tube shunt placement. In this study, we sought to determine the prevalence of and risk factors associated with short- and long-term vision loss and recovery with special attention to unexplained long-term vision loss after aqueous tube shunt surgery. Our study looked specifically at Baerveldt implant (Abbott Medical Optics, Abbott Park, IL), which is one of the two most commonly used types of glaucoma drainage devices. Baerveldt implants are silicone, nonvalved shunts, which require placement of a dissolvable or removable suture around the tube or placement of the plate and tube separately in a two-staged procedure.

While it has been highly debated in the literature whether snuff-out truly exists after trabeculectomy, to our knowledge, no prior studies have explored this phenomenon after aqueous shunt surgery. This is the first study to note the prevalence of decreased vision after tube placement, with the distinction made between transient vision loss and recovery versus long-term vision loss, as well as mild-moderate versus severe vision loss, and to identify risk factors associated with long-term, unexplained vision loss.

## 2. Materials and Methods

We retrospectively reviewed all 350 mm^2^ Baerveldt implantations performed at the University of Southern California (USC) Eye Institute, Keck School of Medicine, Los Angeles, between January 1998 and May 2011, as well as the Massachusetts Eye and Ear Infirmary, Harvard Medical School, Boston, between November 2005 and January 2012. The Institutional Review Boards at USC and Harvard University approved the study protocol, and all study procedures were compliant with the Health Insurance Portability and Accountability Act and the Declaration of Helsinki for research involving human participants.

Inclusion criteria were a minimum of 6-month follow-up period, baseline visual acuity (VA) of counting fingers or better, and one of the following glaucoma diagnoses: primary or secondary open-angle, chronic angle-closure, pseudoexfoliation, pigmentary, traumatic, low-tension, juvenile, or iridocorneal endothelial syndrome. Exclusion criteria were aphakia, other concurrent surgical procedures, or a diagnosis of neovascular, congenital, or uveitic glaucoma. The following preoperative characteristics were noted: age, sex, race, lens status, diagnosis, history of prior aqueous shunt surgery, IOP, Snellen VA, cup-to-disc ratio, Humphrey visual field (HVF) mean deviation, presence of split fixation based on HVF testing, and number of quadrants with split fixation.

We defined split fixation in the same way as our prior study on vision loss after trabeculectomy: a sensitivity of less than 10 dB involving any paracentral points in the four cardinal quadrants (superotemporal, inferotemporal, superonasal, and inferonasal) on 24-2 HVF examination [4]. Follow-up data was obtained at postoperative intervals of 1 day, 1 week, 1 month, 3 months, 6 months, and 12 months, then yearly thereafter, making note of VA, IOP, postoperative procedures, and complications, including choroidal effusions, flat or shallow anterior chamber, and hypotony (IOP ≤ 5). Follow-up duration was determined as the length of time from surgery to the last follow-up visit documented. For two-staged Baerveldt implantations, preoperative data was obtained according to Stage I date and postoperative data according to Stage II date.

Data were evaluated for documentation of postoperative vision loss, which was categorized as mild-to-moderate versus severe. Mild-to-moderate vision loss was defined as a decrease of 3 to 5 lines in Snellen VA compared with preoperative, baseline VA. Severe vision loss was defined as a decrease of more than 5 lines in Snellen VA or semiquantitative categories of low vision (e.g., counting fingers at a given distance, with 7–10 feet, 4–6 feet, and 1–3 feet each approximating one line of Snellen VA; hand motion; light perception and no light perception) compared to baseline VA. The determination of mild-to-moderate versus severe vision loss was based on the lowest observed VA within the 6-month postoperative period.

Vision loss was then categorized as long-term versus transient. Postoperative vision loss was considered long-term if Snellen VA did not recover to within 3 lines of the preoperative VA by the 6-month follow-up period. Conversely, postoperative vision loss was considered transient if there was a return in vision to within 3 lines of the preoperative VA at the 6-month follow-up interval, with note made of the number of days for visual recovery. The postoperative course was reviewed in all cases of long-term vision loss to identify any clinical findings or occurrences that accounted for the vision loss. Cases of severe, long-term vision loss without any identifiable explanation were further characterized as “snuff-out.”

Statistical analysis was performed with STATA 13.1 for Windows (StataCorp, College Station, TX). All descriptive statistics were reported as mean ± standard deviation. Statistical significance was defined as *p* ≤ 0.05, unless multiple comparisons were conducted, in which case the Bonferroni correction was applied.

Preoperative and postoperative variables were compared between patients with long-term, unexplained vision loss and all others using logistic regression analysis. Entry into the initial model was determined via univariate regression based on *p* ≤ 0.25. Elimination proceeded one variable at a time, with each iteration of the model tested for correct specification using the Box-Tidwell test. Nested model iterations were compared using the Bayesian Information Criterion (BIC) and likelihood-ratio test, and elimination proceeded until the BIC indicated no further improvements. Each model was then evaluated for influential observations and data entry errors through inspection of Pearson residuals, deviance residuals, and Pregibon leverages. Robustness of model fit was evaluated through difference of chi-squares and deviances.

## 3. Results

A total of 247 eyes of 222 patients were included in the study. All eyes underwent Baerveldt tube shunt placement without reported intraoperative complications or concomitant surgical procedures.  Table 1 summarizes the patient demographic and preoperative data. The average patient age was 70.1 ± 14.7 years. Females comprised 49.4% of patients. The most prevalent glaucoma diagnoses were primary open angle (60%) followed by chronic angle closure (16%). Seventy-seven percent of patients were pseudophakic. Thirty-one patients (13%) had aqueous shunt placement in the ipsilateral eye previously.

A majority of patients demonstrated evidence of advanced glaucomatous disease. Over 73% had a preoperative cup-to-disc ratio ≥0.9, and the average mean deviation on HVF 24–2 was −15.2 ± 8.7 dB. Fifty-one percent of all preoperative visual fields (95 of 186 HVFs) had split fixation in at least 1 cardinal quadrant.

**Figure 1:**
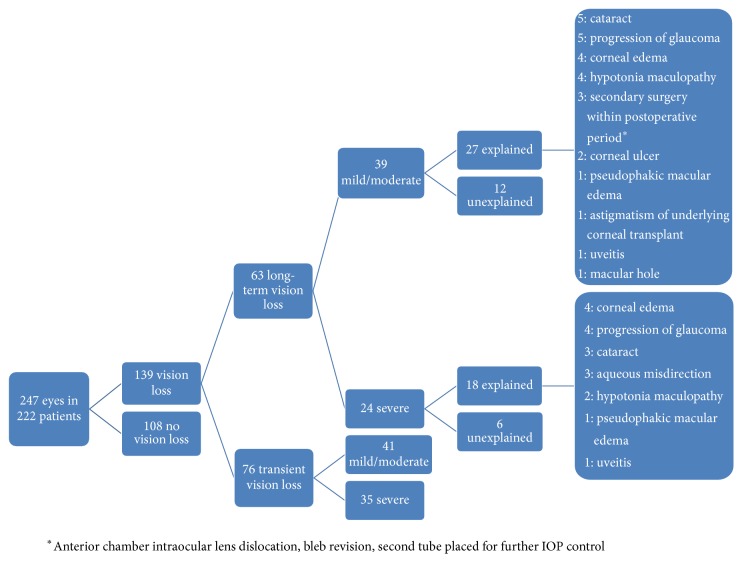
Incidence of vision loss among 247 eyes in 222 patients six months after Baerveldt glaucoma tube shunt implantation.

Figure 1 shows the categorical occurrences of vision loss. 108 of 247 eyes (43.7%) maintained vision within two Snellen lines over the 6-month postoperative period. 76 of 247 eyes (30.8%) had transient vision loss, of which 41 eyes (53.9%) had mild-to-moderate vision loss (3–5 lines of Snellen VA loss from baseline) and 35 eyes (46.1%) had severe loss (>5 lines of Snellen VA). The mean time to recovery for eyes with transient vision loss was 73.3 ± 51.8 days (range 23.5 to 176).

Long-term vision loss was observed in 63 of 247 eyes (25.5%). Thirty-nine of these cases (61.9%) were mild-to-moderate, and 24 (38.1%) were severe. Long-term vision loss could be explained in 45 cases (71.4%). The most common causes were progression of glaucoma, cataract, and corneal edema, accounting for 9, 8, and 8 cases, respectively. All other attributable causes of long-term vision loss are listed in Figure 1. Eighteen cases of long-term vision loss (7.3% of cases overall) had no identifiable explanation. Specifically, 6 cases, or 2.4% of all study eyes, had severe, long-term, unexplained vision loss and were therefore considered cases of snuff-out. Of note, none of the 18 eyes with unexplained long-term vision loss were from the same patient.

Preoperative and postoperative characteristics were compared between cases of unexplained long-term vision loss and all other cases (Table 2). Logistic regression analysis revealed that the only factor significantly associated with unexplained long-term vision loss overall was postoperative day 1 (POD1) Snellen VA (OR = 1.29, 95% CI [1.08–1.55], and *p* = 0.005) (Table 3). The presence of split fixation in the inferonasal quadrant on preoperative HVF was nearly significant (OR = 3.28, 95% CI [0.90–11.93], and *p* = 0.072). Factors significantly associated with snuff-out were the change in preoperative versus POD1 Snellen VA (OR = 1.51, 95% CI [1.06–2.13], and *p* = 0.021) and the presence of split fixation in the inferonasal quadrant on preoperative HVF (OR = 13.70, 95% CI [1.08–17.07], and *p* = 0.044). No other variables examined reached statistical significance in the final multivariate regression analysis.

## 4. Discussion

Our findings suggest that vision decrease is a common occurrence postoperatively and that snuff-out, though uncommon, does occur after aqueous tube shunt implantation. To the best of our knowledge, no prior reports specifically explored the course of and risk factors associated with snuff-out after aqueous tube shunt surgery. However, several studies have described visual acuity outcomes in general after Baerveldt implantation, as summarized in Table 4 [5–8, 10, 11, 13–17].

These previous studies often sought to compare the efficacy of Baerveldt shunts with trabeculectomy or alternative shunt types. They only compared VA at baseline with a single time point after aqueous shunt surgery, with no comments made regarding the course of vision loss and recovery. Moreover, these studies had varying follow-up periods with notable differences in inclusion and exclusion criteria, such that a direct comparison of results between these studies and the present one is not possible. Nevertheless, these prior studies reported an overall mean reduction in logMAR Snellen VA ranging from 0.16 units to 1.6 units (mean of 0.53) with a follow-up period ranging from one to five years after Baerveldt implantation. When visual acuities in this study were converted into their logMAR equivalents, the overall mean reduction in VA was 0.16 units ± 0.49, which is comparable to the aforementioned studies.

A few studies specified the degree of vision loss or gain based on the change in Snellen VA from baseline to postoperative period. Christakis et al. looked at 114 eyes in 114 patients who were randomly assigned to Baerveldt shunt placement and, after three years of follow-up, found that approximately 18% of patients lost 3-4 lines of Snellen VA and approximately 23% lost ≥ 5 lines of Snellen VA [8, 9]. This subdivision is comparable to our definition of mild-to-moderate and severe vision loss, which we defined as 3–5 lines of Snellen VA and >5 lines of Snellen VA from baseline, respectively. At 15.8%, our rate of long-term mild-to-moderate vision loss was similar to the 18% reported by Christakis et al. However, our rate of severe long-term vision loss of 9.7% was significantly less than the 23% that Christakis et al. reported. Given that our minimum follow-up period was six months compared with three years in the previous study, this discrepancy in severe vision loss may be due to patients losing vision over time from progression of their underlying glaucoma. Christakis et al. did not address reasons for vision loss in their study.

Two other study series distinguished a loss of 2 or more Snellen lines after Baerveldt placement, with rates ranging from 30% to 46% over a follow-up period of one to five years [5–7, 10–12]. This degree of vision loss (≥2 lines Snellen VA) is nearly equivalent to the sum of our mild-to-moderate and severe vision loss cases, or 25.5% of all cases, in the current study. Our rate of long-term vision loss may be lower than those previously reported since these studies included patients with a 2-line reduction in Snellen VA, whereas our definition of vision loss started with 3 or more Snellen lines lost. In addition, many prior studies included eyes with neovascular and uveitic glaucoma, which were excluded from the present study, given the high failure rates and overall poor visual outcomes. Furthermore, endpoints of these studies were later than our six months of follow-up, after which patients may suffer vision loss due to glaucomatous progression.

The Tube Versus Trabeculectomy (TVT) and Ahmed Baerveldt Comparison (ABC) studies attempted to identify causes of vision loss [5–7, 10–12]. In the TVT studies, Gedde et al. identified postoperative complications, specifically persistent corneal edema and choroidal effusions, as independent risk factors for vision loss 1 year after Baerveldt placement. This was not explored in subsequent studies. The authors cited progression of glaucoma, macular disease, and cataracts as the most common reasons for vision loss, with 7.2% of the Baerveldt cohort suffering vision loss for unknown reasons at five-year follow-up. This was similar to the 7.3% occurrence of unexplained long-term vision loss in our study, with progression of glaucoma, corneal edema, cataracts, and hypotony maculopathy accounting for the four most common explanations of long-term vision loss.

In the ABC studies, Barton et al. separated patients into 4 strata: Stratum 1: primary glaucoma with previous surgery, Stratum 2: secondary glaucoma (excluding neovascular and uveitic glaucoma), Stratum 3: neovascular glaucoma, and Stratum 4: uveitic glaucoma [18]. At one-year follow-up, the authors found certain diagnostic strata (namely, neovascular glaucoma and “high-risk strata”) and better preoperative VA to be highly predictive of VA loss of 2 or more Snellen lines. This was not addressed in subsequent studies. Neovascular and uveitic glaucoma were excluded in the present study so a direct comparison with these results is not possible. The authors also found progression of glaucoma, macular disease, and cataract to be the most frequent causes of vision loss after Baerveldt placement, with unexplained vision loss occurring in 15% of patients in the overall study population. In contrast with the findings from Gedde et al., postoperative complications were not statistically associated with vision loss.

While these prior studies explored reasons for vision loss, this paper sought to identify and predict* unexplained* vision loss. While this has not been characterized with respect to aqueous shunt surgery, several published studies have explored the risk of snuff-out after trabeculectomy [4, 19–24]. Francis et al. published the most recent study exploring vision loss and snuff-out after trabeculectomy [4]. Among 301 eyes in 262 patients, the authors reported a 2.0% rate of snuff-out after trabeculectomy. While this focuses on a different type of glaucoma surgery, interestingly, we found a similar rate of snuff-out in the present study of 2.4%. On univariate analysis, Francis et al. found that risk factors for long-term vision loss were the presence of split fixation on preoperative HVF, the number of cardinal quadrants with split fixation, and postoperative choroidal effusions with eventual resolution. A limitation of the prior study was the lack of multivariate analysis. Our current multivariate analysis revealed that, of the variables that Francis et al. studied, only the presence of split fixation in the inferonasal quadrant was statistically significant for snuff-out after Baerveldt implantation.

One speculation as to why split fixation may be a risk factor for snuff-out is the fact that fixation is supplied by the maculopapular fiber bundle, which is often a late portion of retinal nerve fiber layer to be compromised in glaucomatous optic nerve damage. Therefore, the presence of split fixation may suggest that the underlying glaucoma is often so advanced that loss of any remaining fixation points may occur more easily, especially after a traumatic event such as glaucoma surgery, and thereby lead to snuff-out postoperatively.

Limitations of this study include the flaws inherent in a retrospective study, including the nonrandomization of patients and the lack of regular HVF examinations. The decision to perform tube shunt placement was made by the treating physician on the basis of overall patient status but was not strictly standardized. Of the 247 eyes included in this study, only 186 eyes had undergone preoperative HVF testing. Visual fields were not performed in the remaining 61 patients due to poor preoperative visual acuity (≤20/200) and/or excessively high IOP necessitating urgent tube placement. In addition, because snuff-out is an uncommon phenomenon, it was necessary to include both eyes from some patients to gain proper statistical power for this retrospective review. However, none of the 18 eyes with unexplained long-term vision loss were from the same patient. Moreover, there was no statistically significant difference in the results when only one eye of these patients was randomly selected for the analysis. Finally, there may have been a bias toward underestimating the incidence of snuff-out if surgeons suspected this possibility in patients with advanced vision loss and therefore did not operate with equal frequency on these patients.

In conclusion, our findings suggest that transient vision loss is common and takes an average of 2.5 months to recover following Baerveldt placement. Long-term vision loss occurs less commonly but still comprises a significant proportion of patients and should thus be included in patient education about the risks and benefits of surgery. Snuff-out, or severe unexplained long-term vision loss, was an uncommon phenomenon, occurring in 2.4% of cases after Baerveldt implantation. Poorer POD1 VA may herald a worse prognosis in the long term, and the level of visual field loss, especially the presence of inferonasal split fixation on preoperative HVF, may help identify patients at the highest risk for snuff-out. The results of this study aim to elucidate the course of vision loss and recovery after Baerveldt placement and to better identify risk factors for unexplained long-term vision loss and snuff-out.

## 5. Conclusions

This is the first study that seeks to explore the phenomenon of snuff-out, or unexplained severe long-term vision loss, after aqueous tube shunt placement. Predictive risk factors for snuff-out are the degree of vision loss on POD1 and the presence of split fixation involving the inferonasal quadrant on preoperative visual field testing. Long-term vision loss is not uncommon after Baerveldt tube shunt implantation, with snuff-out occurring in 2.4% of cases in this study.

## Figures and Tables

**Table 1:** Baseline preoperative demographics of 222 patients undergoing Baerveldt glaucoma tube shunt implantation in 247 eyes.

Demographic	Value (*n* = 247)
Age, *mean (SD), years*	70.1 (14.7)
Follow-up period, *mean (SD), months*	29.1 (22.8)
Sex, *number (%)*	
Male	125 (50.6)
Female	122 (49.4)
Race/ethnicity, *number (%)*	
White	100 (40.5)
Asian	38 (15.4)
Hispanic	45 (18.2)
African American	29 (11.7)
Others	35 (14.2)
Diagnosis, *number (%)*	
Primary open-angle glaucoma	148 (59.9)
Chronic angle-closure glaucoma	40 (16.2)
Secondary open-angle glaucoma	17 (6.9)
Low-tension glaucoma	3 (1.2)
Pseudoexfoliation glaucoma	17 (6.9)
Pigmentary glaucoma	3 (1.2)
Juvenile glaucoma	18 (7.3)
Plateau iris glaucoma	1 (0.4)
Snellen visual acuity, *median (range)*	20/60 (20/15 to counting fingers at 1 feet)
Lens status, *number (%)*	
Phakic	58 (23)
Pseudophakic	189 (77)
Prior filtration surgery, *number (%)*	
Yes	31 (13)
No	216 (87)
Preoperative intraocular pressure	
Mean (SD), mm Hg	24.6 (8.1) (range 11 to 58)
<21, *number (%)*	91 (36.8)
≥21, *number (%)*	156 (63.2)
Cup-to-disc ratio	(*n* = 231)
Mean (SD)	0.88 (0.13) (range 0.3 to 1.0)
<0.9, *number (%)*	62 (26.8)
≥0.9, *number (%)*	169 (73.2)
Humphrey visual field mean deviation	(*n* = 175)
Mean (SD), dB	−15.2 (8.7) (range −32.7 to 10.5)
<−12, *number (%)*	102 (58.3)
≥−12, *number (%)*	73 (41.7)
Preoperative split fixation on visual fields, *number (%)*	(*n* = 186)
Yes	95 (51.1)
No	91 (48.9)
Location of quadrants with split fixation, *number (%)*	(*n* = 186)
Inferonasal	42 (22.6)
Inferotemporal	39 (21.0)
Superonasal	66 (35.5)
Superotemporal	59 (31.7)
Quadrants with split fixation, *number (%)*	(*n* = 186)
Mean (SD)	1.1 (1.4)
0	91 (48.9)
1	36 (19.4)
2	28 (15.1)
3	10 (5.4)
4	21 (11.3)

**Table 2:** Descriptive characteristics of unexplained long-term vision loss among 247 eyes after Baerveldt tube shunt implantation.

Variable	Long-term unexplained vision loss			
	Mild or moderate	Severe	Total	Others
	(*n* = 12)	(*n* = 6)	(*n* = 18)	(*n* = 229)
Age, *mean (SD), years*	75.8 (10.2)	63.1 (24.1)	71.6 (16.7)	70.0 (14.5)
Sex^Ø^, *number (%)*				
Male	5 (41.7)	6 (100)	11 (61.1%)	114 (49.8)
Female	7 (58.3)	0	7 (38.9%)	115 (50.2)
Race/ethnicity, *number (%)*				
White	10 (83.3)	1 (16.7%)	11 (61.1)	89 (38.9)
Asian	1 (8.3)	1 (16.7%)	2 (11.1)	36 (15.7)
Hispanic	1 (8.3)	2 (33.3%)	3 (16.7)	42 (18.3)
African American	0	1 (16.7%)	1 (5.6)	28 (12.2)
Others	0	1 (16.7%)	1 (5.6)	34 (14.8)
Diagnosis^†^, *number (%)*				
Primary open-angle glaucoma	8 (66.7)	4 (66.7)	12 (66.7)	136 (59.4)
Chronic angle-closure glaucoma	1 (8.3)	0	1 (5.6)	40 (17.5)
Secondary open-angle glaucoma	1 (8.3)	0	1 (5.6)	15 (6.6)
Low-tension glaucoma	0	0	0	3 (1.3)
Pseudoexfoliation glaucoma	2 (16.7)	0	2 (11.1)	15 (6.6)
Pigmentary glaucoma	0	1 (16.7)	1 (5.6)	2 (0.9)
Juvenile glaucoma	0	1 (16.7)	1 (5.6)	17 (7.4)
Plateau iris glaucoma	0	0	0	1 (0.4)
Preoperative Snellen visual acuity, *median (range)*	20/70 (20/20 to 20/400)	20/75 (20/50 to 20/200)	20/75 (20/20 to 20/400)	20/60 (20/15 to 20/4000)
Postoperative day 1 (POD1) Snellen visual acuity^†‡^, *median (range)*	20/800 (20/70 to LP)	20/400 (20/200 to LP)	20/400 (20/70 to LP)	20/200 (20/25 to HM)
Change in preoperative versus POD1 Snellen visual acuity^†‡^, *mean lines Snellen VA (SD)*	5.8 ± 3.9	6.6 ± 4.2	6.1 ± 3.9	3.1 ± 3.6
Lens status^†^, *number (%)*				
Phakic	2 (16.7)	3 (50)	5 (27.8)	53 (23.1)
Pseudophakic	10 (83.3)	3 (50)	13 (72.2)	176 (76.9)
Preoperative intraocular pressure^†^, *mean (SD), mmHg*	22.7 ± 6.1	32 ± 13.5	25.8 ± 9.9	24.5 ± 8.0
Preoperative intraocular pressure				
<21, *number (%)*	5 (41.7)	2 (33.3)	7 (38.0)	84 (36.7)
≥21, *number (%)*	7 (58.3)	4 (66.7)	11 (61.1)	145 (63.3)
POD1 intraocular pressure, *mean (SD), mmHg*	23.1 ± 12.2	20.7 ± 11.8	22.2 ± 11.7	20.2 ± 11.9
Change in preoperative versus POD1 intraocular pressure^†^, *mean (SD)*	0.2 ± 4.2	−11.3 ± 20.7	−4.1 ± 16.1	−4.2 ± 13.6
Cup-to-disc ratio				(*n* = 213)
Mean (SD)	0.9 ± 0.06	0.93 ± 0.06	0.91 ± 0.06	0.87 ± 0.13
Cup-to-disc ratio				
<0.9, *number (%)*	3 (25)	1 (16.7)	4 (22.2)	58 (27.2)
≥0.9, *number (%)*	9 (75)	5 (83.3)	14 (77.8)	155 (72.8)
Humphrey visual field mean deviation, *mean (SD), dB*	(*n* = 9)	(*n* = 2)	(*n* = 11)	(*n* = 164)
	−16.5 ± 7.0	−16.8 ± 17.0	−16.6 ± 8.3	−15.1 ± 8.7
Humphrey visual field mean deviation, *dB*				
<−12, *number (%)*	6 (66.7)	1 (50)	7 (63.6)	95 (57.9)
≥−12, *number (%)*	3 (33.3)	1 (50)	4 (36.4)	69 (42.1)
Preoperative split fixation on visual fields^‡^, *number (%)*	(*n* = 10)	(*n* = 4)	(*n* = 14)	(*n* = 172)
Yes	8 (80)	3 (75)	11 (78.6)	84 (48.8)
No	2 (20)	1 (25)	3 (21.4)	88 (51.2)
Location of quadrants with split fixation^†‡^, *number*				
Inferonasal	4	3	7	35
Inferotemporal	4	2	6	33
Superonasal	6	3	9	57
Superotemporal	5	2	7	52
Quadrants with split fixation^†‡^, *number (%)*				
0	2 (20)	1 (25)	3 (21.4)	88 (51.2)
1	3 (30)	0	3 (21.4)	33 (19.2)
2	2 (20)	1 (25)	3 (21.4)	25 (14.5)
3	0	0	0	10 (5.8)
4	3 (30)	2 (50)	5 (35.7)	16 (9.3)
Postoperative choroidal effusions^*Ø∗*^, *number*				
Yes	0	0	0	26 (11.4)
No	12 (100)	6 (100)	18 (100)	203 (88.6)

† indicates variable met inclusion criteria for the model for snuff-out (severe unexplained permanent vision loss).

‡ indicates variable met inclusion criteria for the model for unexplained permanent vision loss (including mild-to-moderate and severe types).

Ø indicates variable was perfectly predictive of snuff-out.

*∗* indicates variable was perfectly predictive of unexplained permanent vision loss.

HM = hand motion visual acuity.

LP = light perception only visual acuity.

**Table 3:** Multivariate analyses for risk factors predicting unexplained long-term vision loss.

	Long-term unexplained vision loss	
	Severe	Total
Number obs.	166	161
Constant	0.001	0.002
	[0.000–0.030]^*∗∗∗*^	[0.000–0.027]^*∗∗∗*^
Split fixation in the inferonasal quadrant	13.703	3.276
	[1.079–17.071]^*∗*^	[0.899–11.934]
Change in preoperative versus postoperative day 1 Snellen visual acuity	1.505	—
	[1.064–2.128]^*∗*^	—
POD1 VA	—	1.294
	—	[1.083–1.548]^*∗∗*^
Pseudo-*R*^2^	0.315	0.207
Likelihood ratio *χ*^2^	17.70^*∗∗∗*^	11.89^*∗∗*^

Coefficients are presented as odd ratios. 95% confidence intervals for odds ratios appear in brackets.

*∗* indicates significance at the 0.05 level.

*∗∗* indicates significance at the 0.01 level.

*∗∗∗* indicates significance at the 0.001 level.

**Table 4:** Prior studies of vision loss after Baerveldt tube shunt implantation.

Paper, year published	Study Design	Patients included in study	Number of eyes/patients & follow-up (f/u)	Major differences in inclusion/exclusion criteria versus the present study	Average vision loss (mean ± SD)	Significant vision loss after Baerveldt implantation	Comments regarding significant vision loss after BVI
Present study	Retrospective study	Medically uncontrolled glaucoma; underwent BVI placement	247 eyes in 222 patients Minimum 6- month f/u	(i) Inclusion criteria: baseline VA ≥ count fingers, OAG, CACG, PXE, pigmentary, traumatic, low-tension, juvenile glaucoma (ii) Exclusion criteria: aphakia, another concurrent surgery, NVG, congenital + uveitic glaucoma	1.23 ± 3.27 Snellen line decrease in VA from baseline to postoperative month 6 (or 0.16 units ± 0.49 units when converted into logMAR equivalents)	(i) 25.5% had long-term vision loss (ii) 7.3% had unexplained long-term vision loss (iii) 2.4% had snuff-out	Risk factors for snuff-out: (1) decreased visual acuity on postoperative day one versus baseline, (2) preoperative split fixation in the inferonasal quadrant on HVF

The Ahmed Baerveldt Comparison (ABC) studies: 2015 [5], 2014 [6], 2011 [7]	Prospective, multicenter, randomized, controlled trial	Medically uncontrolled glaucoma; randomly assigned to Ahmed or BVI placement	133 eyes in 133 patients underwent BVI 117 eyes at 1-year f/u 100 eyes at 3-year f/u 87 eyes at 5-year f/u	(i) Required IOP ≥ 18 mmHg; included NVG, uveitic glaucoma (ii) Excluded cases with prior cyclodestructive procedures, scleral buckle, silicone oil, or aqueous shunt placement in the same eye	Units logMAR Snellen VA decrease from baseline: 0.16 units, or 1.04 (20/219) at baseline to 1.20 (20/317) at 1-year f/u 0.26 ± 0.74 at 3-year f/u 0.43 ± 0.84 at 5-year f/u	% that lost ≥2 Snellen lines VA from baseline: 34% at 1-year f/u (unexplained VA loss in 6 patients, or 5.1%) 30% at 3-year f/u (unexplained VA loss in 1 or 1.0%) 44% at 5-year f/u (unexplained VA loss in 2 or 2.3%)	(i) Diagnostic stratum (NVG + high-risk strata) and baseline VA were significant predictors of ≥2 Snellen line VA loss at 1-year f/u (ii) Postoperative complications were not significant (iii) Most frequent causes of vision loss: glaucomatous progression, corneal edema, retinal disease, cataract (iv) 96% (24/25) of eyes that progressed to NLP VA had NVG

The Ahmed Versus Baerveldt (AVB) Studies: 2013 [8], 2011 [9]	Prospective, multicenter, randomized, controlled trial	≥18 years old; medically uncontrolled or high-risk glaucoma; randomly assigned to Ahmed or BVI placement	114 eyes in 114 patients underwent BVI 105 eyes at 1-year f/u 90 eyes at 3-year f/u	(i) Required uncontrolled glaucoma refractory to maximum medical therapy (ii) Included NVG, uveitic glaucoma	Units logMAR Snellen VA decrease from baseline: 1-year not stated 1.6 ± 1.2 at 3-year f/u	At 3-year f/u: (i) 23% of BVI patients had ≥5 Snellen lines VA loss (ii) 6 (5%) in the BVI group progressed to NLP vision	At 3-year f/u: (i) Of 11 patients (5%) who progressed to NLP after Ahmed or BVI implantation, 7 (64%) had NVG (ii) Did not explore reasons or risk factors for vision loss

The Tube Versus Trabeculectomy (TVT) Studies: 2012 [10], 2009 [11], 2007 [12]	Prospective, multicenter, randomized, controlled trial	Medically uncontrolled glaucoma with previous CE/IOL and/or failed trabeculectomy; randomly assigned to trabeculectomy or BVI placement	107 eyes in 107 patients underwent BVI 97 eyes at 1-year f/u 80 eyes at 3-year f/u 69 eyes at 5-year f/u	(i) Excluded cases with ICE syndrome, severe posterior blepharitis, prior cyclodestructive procedure, scleral buckle, or silicone oil placement	Units logMAR Snellen VA decrease from baseline: 0.42 ± 0.54 at baseline to 0.61 ± 0.75 at 1-year f/u 0.24 ± 0.58 at 3-year f/u 0.38 ± 0.72 at 5-year f/u	% that lost ≥2 Snellen lines VA from baseline: 32% at 1-year f/u (unexplained vision loss in 8 patients, or 8.2%) 31% at 3-year f/u (unexplained VA loss in 4, or 5%) 46% at 5-year f/u (unexplained VA loss in 5, or 7.2%)	(i) Most frequent causes of vision loss were progression of glaucoma, macular disease, cataract (ii) Postoperative complications were significantly higher if lost ≥2 Snellen lines VA (45%) than if not (20%) at 1-year f/u (iii) Persistent corneal edema + choroidal effusions were independent predictors of vision loss at 1-year f/u

Clinical experience with the Baerveldt 250-mm^2^ glaucoma implant [13], 2006	Retrospective study	No prior drainage implants and underwent BVI placement between 3/96 and 12/02	108 eyes in 108 patients Mean f/u of 22.8 ± 20.3 months	(i) Included all types of glaucoma	logMAR Snellen VA decrease: (i) For all types of glaucoma: 1.32 (20/420) at baseline to 1.57 (20/750) post-op (ii) For non-NVG: 0.98 (20/190) at baseline to 1.19 (20/310) post-op	Not addressed	Not addressed

The Ahmed Shunt versus the Baerveldt Shunt for Refractory Glaucoma: A Single-Surgeon Comparison of Outcome [14, 15], 2003, 2006	Retrospective study	Consecutive patients with refractory glaucoma; Ahmed or BVI placement; no concurrent surgeries	70 eyes in 70 patients underwent BVI 4-year f/u	(i) Included uveitic glaucoma, NVG, aphakic patients	(i) 78% of patients had retained or improved VA compared to preoperative VA after BVI placement	2 patients (3%) developed NLP vision in the postoperative period (reasons not specified)	Not addressed

Baerveldt 350-mm^2^ Implant versus Ahmed Valve for Refractory Glaucoma: A Case-Controlled Comparison [16], 2004	Retrospective study	BVI placement with no prior tube or cyclodestruction procedures	32 eyes in 32 patients 1-year f/u	(i) Included NVG, congenital glaucoma, aphakic patients	Not addressed	43.3% lost ≥2 Snellen lines after BVI placement	Not addressed

BVI = Baerveldt implant; CACG = chronic angle-closure glaucoma; CE/IOL = cataract extraction with intraocular lens implantation; f/u = follow-up; NLP = no light perception; NVG = neovascular glaucoma; OAG = open-angle glaucoma; post-op = postoperatively; PXE = pseudoexfoliation; Trab = trabeculectomy; VA = visual acuity.
